# A Polymer Plugging Gel for the Fractured Strata and Its Application

**DOI:** 10.3390/ma11050856

**Published:** 2018-05-21

**Authors:** Xiangyu Fan, Pengfei Zhao, Qiangui Zhang, Ting Zhang, Kui Zhu, Chenghua Zhou

**Affiliations:** 1 College of Petroleum Engineering, Southwest Petroleum University, Chengdu 610500, China; fanxy666@swpu.edu.cn (X.F.); 18081033887@163.com (P.Z.); zt2018330@163.com (T.Z.); 2 State Key Laboratory of Oil and Gas Reservoir Geology and Exploitation, Southwest Petroleum University, Chengdu 610500, China; 3 Department of Civil, Geological and Mining Engineering, École Polytechnique de Montréal, Montreal, QC H3T 1J4, Canada; 4 Sinopec Nanjing Chemical Research Co., Ltd., Nanjing 210000, China; vankung@126.com; 5 Drilling Engineering Research Institute of Sinopec Southwest Petroleum Engineering Co., Ltd., Deyang 618000, China; zhoucheng0117@163.com

**Keywords:** drilling engineering, well leakage, fractured strata, polymer gel, plugging material

## Abstract

Well leakage of fractured strata is a tricky problem while drilling. This unwieldy problem is usually caused by the poor formation of the cementing degree, the staggered-mesh of the fracture, and the low bearing capacity of the formation, which can also lead to a narrow and even unsafe window of drilling fluid density. For fractured strata, the normal plugging material has the disadvantages of unsuitable size and low strength, resulting in unsuccessful first time plugging and an increase in cost. Therefore, we developed a polymer plugging gel for the fractured strata, named XNGJ-3. XNGJ-3 is mainly made of an acrylamide monomer and is accompanied by the reactive monomers of carboxyl and hydroxyl as ingredients. XNGJ-3 has a low viscosity before gelling. At 80 °C it becomes gelled, and the gelling time was controlled within the required time of the practical application. These conditions are beneficial for making the plugging material enter the crossing fracture smoothly and occlude the fracture. XNGJ-3 also has a good deformability and can avoid being damaged during the process of fracture closure. The well leakage simulated experiment revealed that the bearing capacity of this material can reach 21 MPa and the inverse bearing capacity can reach 20 MPa. These strengths are more than twice that of common polymer plugging gels. Finally, three leaked wells in the fractured strata of the Sichuan Basin were used to verify the plugging effect of XNGJ-3. Compared with other common plugging materials, XNGJ-3 has the advantages of having a higher success rate of first time plugging, a lower economic cost, a shorter work time, and so forth, which indicate that this plugging material has a good engineering application value in dealing with well leakage of fractured strata.

## 1. Introduction

Well leakage is a common drilling accident. When it occurs, the drilling fluid enters the strata under a pressure difference, resulting in some complicated situations [1]. Well leakage is more serious during drilling of the fractured strata. This kind of leakage is difficult to handle because of the broken strata, complex fractures, and low bearing capacity of strata. There are many microfractures and disconnected fractures in the initial strata, these fractures do not cause leakage at first, but expand and connect under wellbore pressure, and can eventually lead to well leakage [2,3]. Also, the stratum of interlaced fractures has poor cementation and is very susceptible to pressure [4]. Currently, engineers and researchers in this field have realized that a suitable material would be key to solve well leakages for different kinds of strata, and thus, a lot of plugging materials have been invented [5,6,7,8,9]. The traditional and most common plugging method is bridge plugging, which is when the plugging materials are used as padding and bridging to form a plugged zone with a suitable strength [10,11,12,13,14]. However, the fracture size of the fractured strata changes a lot, which can cause the bridge materials to find it difficult to enter the fracture or to find it hard to form the bridge plugged zone [15,16,17]. This kind of well leakage accident is a really troublesome problem in the downhole, which can result in a large loss in terms of both the economic cost and drilling time, and can even do harm to drilling workers.

Nowadays, gel plugging materials have been widely used in well-plugging areas due to their numerous advantages [18,19,20]. These polymer gel materials, which are composed of many monomers linked by covalent bonds, are usually long-chain molecules. Their chain length is affected by some factors such as polymerization conditions, monomer concentration, and covalent bond strength. The properties of these materials, such as the rheology, solubility, sol-gel changing condition, the strength, and so on, are affected by their structure, the type of functional group, and the reaction monomer concentration [21,22,23,24]. Therefore, a gel material with a special functionality can be used as a plugging material for many kinds of strata by designing the proper molecule. Using the fluidity property of the gel material before gelling, Wang [25] took Konjac flour as a principal ingredient and then adjusted the pH, adhesive, and grafting monomer to control the strength of the plugging gel and finally developed a gel plugging material. Du [26,27] developed a thermoresponsive temporary plugging material which could congeal and dissolve depending on the temperature. Luo [28,29] developed a kind of gel plugging material which has a good fluidity at high fluid velocity and high viscosity at low velocity. This material cannot mix with gas and water and is used in the stratum containing the water layer. Mahmoud and Bai [30,31] analyzed the influence of the permeability of the pressure gel under back pressure and obtained the effects of particle size, back pressure, and other factors on permeability. Although there are many successful applications of the gel plugging material, there are several defects in the present gel. Most of the plugging gels are preformed gels, and still have some limitations for well leakage in fractured strata: (1) The material is sent to the formation in a high viscosity gel state, thus causing it not to enter small fractures [28,29]. (2) The fluidity of the gel is highly influenced by the environment and the material may congeal at inappropriate times resulting in the failure of plugging. (3) The bearing strength of the plugging gel is not good enough for the fractured strata [26,27,32,33], which may cause the plugged zone to fail under positive pressure or back pressure.

Therefore, considering the limitations of the current plugging materials and the characteristics of the fractured strata, in this paper, we developed a polymer plugging gel named XNGJ-3. In addition, the fluidity, mechanical strength, consolidation time, pressure bearing capacity, and plugging effect of this new material were tested in laboratory experiments. Additionally, the practical engineering application was evaluated using a leaking well in the Sichuan basin, southwest China.

## 2. Polymer Plugging Gel 

The polymer plugging gel (XNGJ-3) comprises of the main component and a kind of bridge material. The main component of XNGJ-3 is a kind of soluble polymer gel particle. This polymer gel particle contains a functional group which can polymerize under certain conditions. So, XNGJ-3 can flow into the formation as a liquid and congeal to form an agglomeration in the formation fracture to plug the well leakage. Meanwhile, a bridge material is mixed with the polymer gel particles to increase its strength. The polymer gel particles and the bridge material are introduced as follows.

### 2.1. Polymer Gel Particles

#### 2.1.1. Basic Framework for Preparing the Polymer Gel Particles

For developing the polymer plugging gel, we needed to know the characteristics of the normal plugging material used for the fractured strata, which can be drawn from the following. (1) The bridge plugging materials are difficult to enter the fractured area due to the complex fractures’ size. (2) The bearing capacity of the fractured strata is poor which can cause failure of the leakage area. (3) The reversed bearing capacity of the plugging material is low, resulting in the flowing back of the plugging material into the wellbore during the swabbing operation. For solving these problems, acrylamide was used in this developed plugging material as the main component of the water-solubility monomer, considering its good fluidity before gelling [27,34,35]. Then, carboxyl and hydroxy containing monomers were added to the reaction monomer to make the material congeal under a certain conditions of temperature, pressure, and catalyst type [36,37]. Most importantly, the gel-formation time of the new plugging material needs to meet the requirement of the plugging operation and this plugging material must have enough strength to resist the positive and opposite pressures in the fracture even in high-temperature conditions.

#### 2.1.2. Preparation Method of the Polymer Gel Particles

Raw materials: deionized water, acrylamide A, monomer B (containing carboxyl), monomer C (containing hydroxy), emulsifier agent D, and evocating agent E. All the chemicals are analytical reagents without further purification.

Devices: an autoclave of 5 m^3^ was used for reacting; a high groove of 2 m^3^ was used for reacting; a water circulation pump was used for replacing the gas; a drier was used for drying the emulsion; a comminutor was used for breaking the gel block; a vibration sieve was used for screening the material.

**Figure 1:**
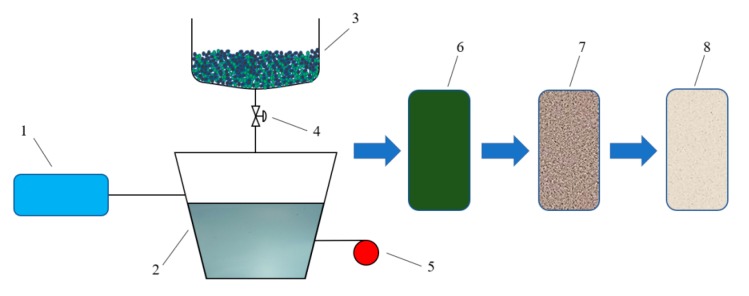
The preparation diagram of the polymer gel particles: 1 is a vacuum pump; 2 is a autoclave; 3 is a high groove; 4 is a valve; 5 is a temperature control instrument; 6 is a and 5 are piston vessels; 6 is a drier; 7 is a comminutor; 8 is a vibration sieve.

**Figure 2:**
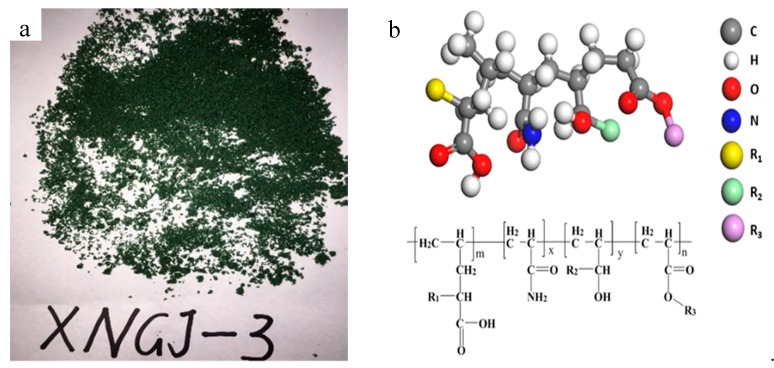
The polymer gel particles. (**a**) A physical photograph; (**b**) a schematic diagram of the molecular structure.

Reaction process: (1) In order to ensure that the polymerization reaction was not disturbed by oxygen and other gases in the air, the reaction pipeline and reactor were fully replaced by a vacuum pump before the reaction, so that the reaction always a nitrogen-rich environment. (2) Emulsifier D was mixed with water to form the micelle liquid in the autoclave, then material A and evocating agent E were added by stirring to form the emulsion. (3) Materials B and C were stirred evenly in the high groove, and then 30% of the mixed monomer in the autoclave was transferred into the autoclave. (4) At autoclave temperature of 30 °C, the rest of the mixed monomer (70%) was transferred into the autoclave within 2 h. Then the No. 2 autoclave was heated to 60 °C at a speed of 0.5 °C/min. After that, the temperature was kept steady until the end of the reaction, which took about 6 h. The mixed monomers became an emulsion. (5) The emulsion was spray dried until reaching the required water moisture level and then the material was crushed when it was reduced to room temperature. After that, a vibration sieve was used to screen the polymer gel particles to guarantee that the solid contents were no less than 90% and the particles would pass through the vibration sieve containing 20–100 holes per one square inch. Figure 1 is the preparation diagram of the polymer gel particles. Figure 2 is the polymer gel particles.

#### 2.1.3. FT-IR Measurements of the Polymer Gel Particles

The products of polymer polymerization are complex, which are greatly influenced by raw materials，temperature，reaction time, and other conditions. To verify that the obtained materials have pre-designed molecular structure, it is necessary to analyze the material. The infrared spectrometer can be used to study the structure and chemical bond of molecules, and thus it is a good method for characterizing and distinguishing the chemical species. So, an infrared spectrometer of NO was used. Nicolet 6700, produced by Thermal Scientific (Mansfield, TX, USA), was used to identify the polymer gel particles. This instrument, which uses a middle-far infrared source, has a measurable range from 7800 to 350 cm^–1^ and its precision was 0.01 cm^–1^. Firstly, the polymer gel particles were mixed evenly with the KBr at a ratio of 1:200 (the polymer gel particles: the KBr), and then, this mixed material was made flaky under the pressure of 15 MPa. Finally, the flakiness was tested by the infrared spectrometer.

**Figure 3:**
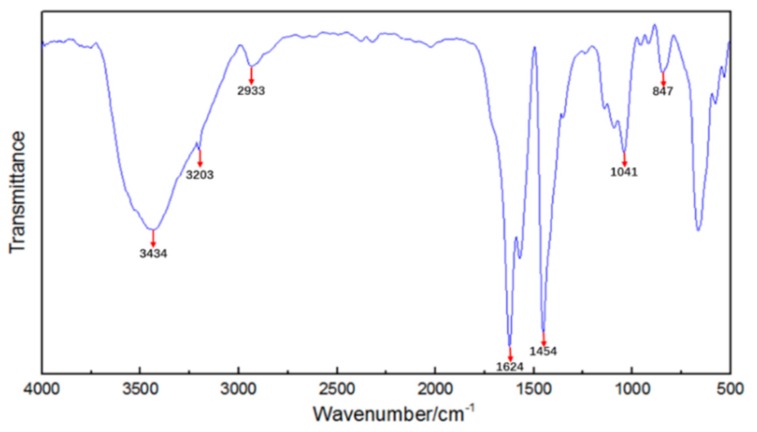
The FT-IR spectrum of the polymer gel particles.

Figure 3 is the result of FT-IR (flourier transformation infrared spectroscopy) measurements of the polymer gel particles. The relationship between wavenumber and transmittance is illustrated in the handbook for use of FT-IR measurements instrument and Analytical Chemistry [38]. The O-H stretching vibration peak in the alcohol hydroxyl group was 3434 cm^–1^. The N-H stretching vibration peak in the amino group was 3203 cm^–1^. The C-H stretching vibration peak in the alkyl was 2933 cm^–1^. The C=O stretching vibration peak was 1624 cm^–1^. The bending vibration peak was 1454 cm^–1^. The O-C=O stretching vibration peak in the ester was 1041 cm^–1^. The stretching vibration peak of the asymmetric ether bond C-O-C was 847 cm^–1^. These indicated that the polymer gel particle that was made using the method introduced in Section 2.1.2 can match the required molecular structure as shown in Figure 2b.

### 2.2. Bridge Material

For meeting the plugging request of the fractured strata, we added a fiber bridge material to increase the strength of the polymer plugging gel (XNGJ-3). This fiber bridge material has the following advantages. (1) The fiber is able to disperse well in the prepared solution because of its lightweight. (2) Comparing with other bridge material, such as the particle and platy material, the fiber has the characteristics of elasticity and flexibility, which can adapt to complex fractures. (3) The fiber can also take the shape of a physical net structure accompanying the net structure of the gel, which can enhance the gel-formation strength and viscous force of the gel plugging material.

The dispersion stability is very important for the bridge material. The normal polyolefin fiber has characteristics of hydrophobicity, so it needs to select a variety of hydrophilic modifications to enhance the dispersion stability of the material in the plugging mud. The interaction of the polyolefin and polymer plugging material was evaluated here, as well as its distribution in the basal body.

**Figure 4:**
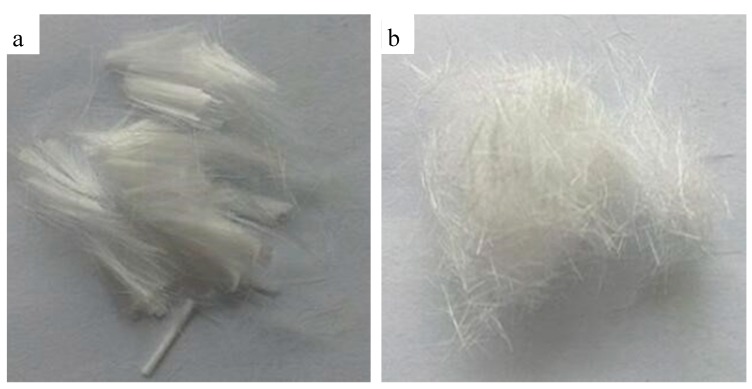
The normal polyolefin fiber (**a**) and hydrophilic fiber (**b**).

**Figure 5:**
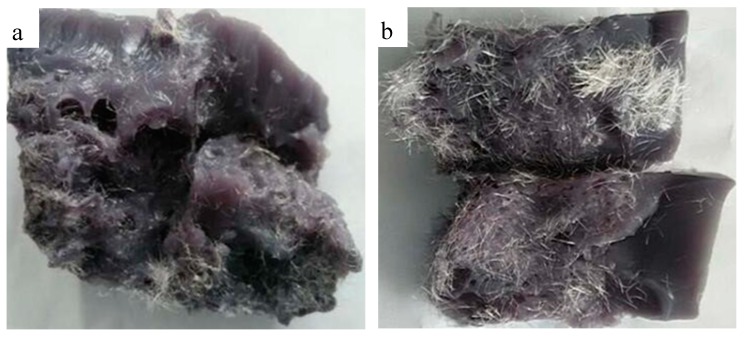
The distribution of the normal polyolefin fiber (**a**) and the distribution of the hydrophilic fiber (**b**).

Figure 4 presents the normal polyolefin fiber and hydrophilic fiber and Figure 5 presents the distribution of the normal polyolefin fiber and hydrophilic fiber in the basal body. The normal polyolefin fiber disperses unevenly in the basal body and the interaction between the fiber and the basal body is weak, so the enhancing action is limited. After the surface hydrophilic modification, it disperses in the basal body uniformly and the interaction between the modified fiber and the basal body is strong. This modified fiber has a strong bridging effect, so the hydrophilic fiber was used as the bridge material in XNGJ-3. 

## 3. Function Evaluation of XNGJ-3

### 3.1. Fluidity Evaluation

#### 3.1.1. Experiment Device and Method

**Figure 6:**
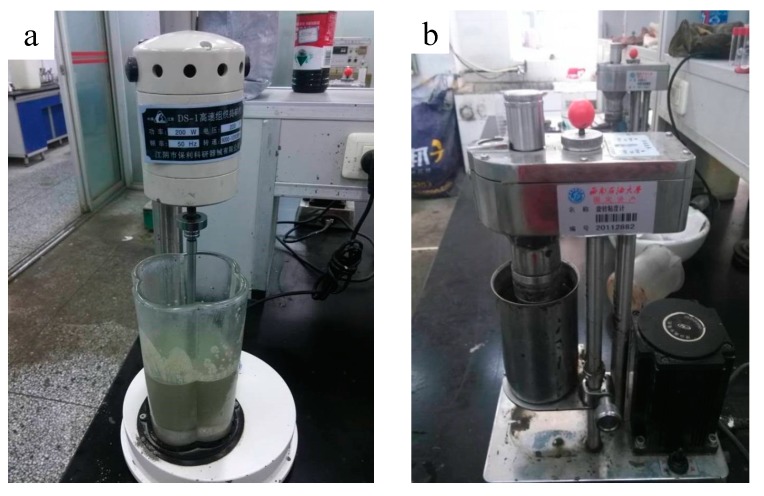
The sample of XNGJ-3 (**a**) and the six-speed rotating viscometer (**b**).

A good fluidity of a plugging material before consolidation can make it enter fractures smoothly. In order to test the fluidity of XNGJ-3, a six-speed rotating viscometer (Heng Taida Co. Ltd., Qingdao, China) was used. 5% bentonite and 10.3% XNGJ-3 (10% polymer gel particles and 0.3% bridge material of the modification fiber, all ratios are by weight and the following is the same) were mixed within the water to form the sample. Figure 6 shows the sample and the six-speed rotating viscometer.

#### 3.1.2. Experiment Results

In practical engineering, the plugging slurry would not be placed very long before its use, so we tested the fluidity within 3 hours after preparation. Table 1 shows the experimental results.

The experimental results show that the plastic viscosity, dynamic shearing force, and static shearing force of the gel solution are very low. Therefore, the gel solvent has a good fluidity and can enter the complex fractures smoothly in the actual plugging process.

### 3.2. Evaluation of the Coagulation Time

The coagulation time of a plugging gel is an important parameter for the plugging material selection. A good plugging gel material must easily flow into the fractures and congeal at the right time. 

#### 3.2.1. Experiment Device and Method

**Figure 7:**
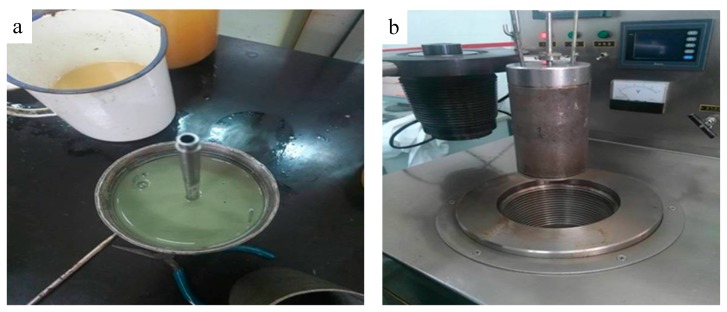
The high temperature and pressure thickener of No. OWC-9380: (**a**) the reaction vessel; (**b**) the operating floor of the device.

The high temperature and pressure thickener (Institute of Applied Technology of Shenyang Aerospace University, Shenyang, China) was used for evaluating the coagulation time of XNGJ-3, as shown in Figure 7. Its work pressure can reach 200 MPa and the work temperature can be 250 °C. This device includes the monitoring instruments for pressure, temperature, and consistency. All of the measured data were recorded by a computer. 

According to standard engineering practice, the coagulation time of XNGJ-3 should be within 90–160 min to meet the requirements of the field plugging operation. So, 5% bentonite and 10.3% XNGJ-3 (10% polymer gel particles and 0.3% bridge material of the modification fiber) were mixed within the water to form the sample. The test method was as follows. (1) The mixed material was put into the reaction vessel and was heated by oil-bath under a closed environment. (2) A rotating bar in the reaction device, which was connected with a consistency testing instrument, was rotated to measure the revolving resistance and to evaluate the consistency and coagulation time. 

#### 3.2.2. Experiment Results

According to the actual situation of the strata, these tests were conducted in 5 different conditions of temperatures and pressures; the coagulation time of XNGJ-3 was also adjusted by the coagulant and retarder. Table 2 presents the test conditions and the results, and Figure 6 presents the coagulation time curves of XNGJ-3 under different conditions.

**Figure 8:**
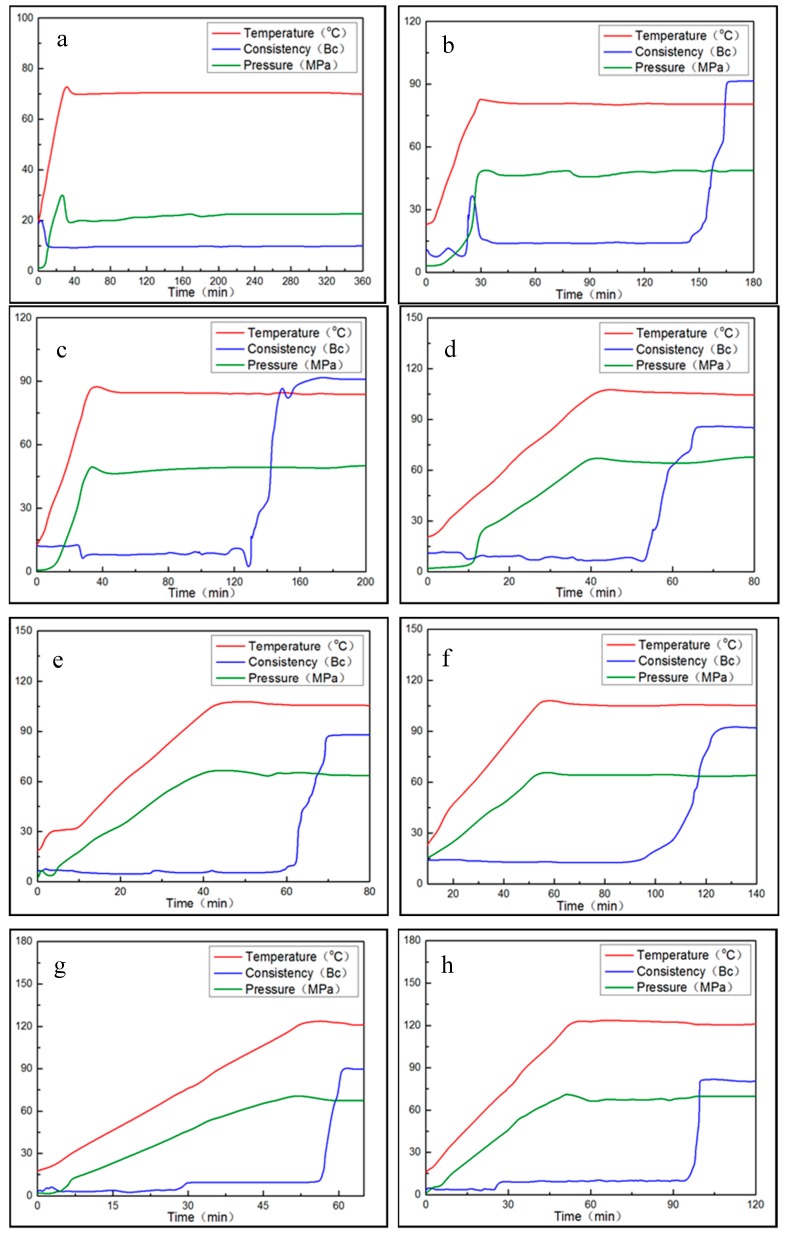
The coagulation time curves of XNGJ-3 under different conditions: (**a**) under the conditions of 70 °C, 20 MPa, 2% coagulant; (**b**) under the conditions of 80 °C, 20 MPa, 1% coagulant; (**c**) under the conditions of 90 °C, 50 MPa; (**d**) under the conditions of 105 °C, 65 MPa, 0.2% retarder; (**e**) under the conditions of 105 °C, 65 MPa, 0.4% retarder; (**f**) under the conditions of 105 °C, 65 MPa, 0.6% retarder; (**g**) under the conditions of 120 °C, 80 MPa, 0.6% retarder; and (**h**) under the conditions of 120 °C, 80 MPa, 0.8% retarder.

**Figure 9:**
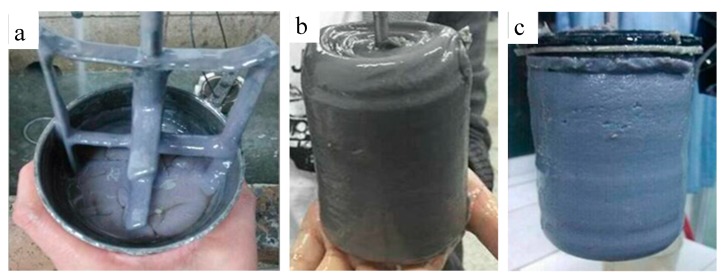
The coagulation characteristics of XNGJ-3 under different conditions: (**a**) under the conditions of 70 °C, 20 MPa, 2% coagulant, not consolidated; (**b**) under the conditions of 90 °C, 50 MPa, consolidated after 144 min; and (**c**) under the conditions of 120 °C, 80 MPa, 0.8% retarder, consolidated after 97 min.

The following conclusions can be drawn from the Figure 8: (1) The consistency of XNGJ-3 is very low before reaching the primary solidification point which indicates that the material has a good fluidity and that it is beneficial for flowing into the fracture. (2) While the temperature of the environment is below 80 °C, XNGJ-3 does not gel even if a large amount of coagulant is added. The reason for this is because the temperature does not reach the coagulation point of XNGJ-3. However, when the temperature is 80 °C, the XNGJ-3 can gel after 154 min and the primary coagulation time becomes shorter with an increase in temperature and pressure. (3) The initial coagulation time of XNGJ-3 is between 90 and 160 min can be adjusted by adding a retarder or coagulant to fit the requirement of the coagulation time in the practical plugging operation. (4) The whole gelling process of XNGJ-3 was controlled over 15 min and this process will be shorter with the increase of the temperature and pressure, which is good for plugging the fracture and reducing the loss of the plugging material. Figure 9 presents the non-congealed material (a) and congealed material (b and c) after the tests.

### 3.3. Uniaxial Compression Experiment

#### 3.3.1. Experiment Device and Method

**Figure 10:**
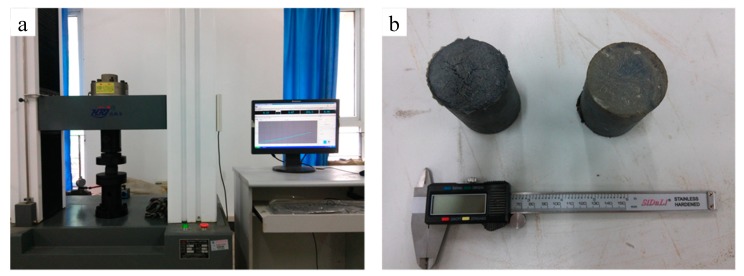
The uniaxial compression apparatus (**a**) and the cylinder gel blocks (**b**).

In order to evaluate the strength of material, XNGJ-3 was tested by uniaxial compression apparatus (Hengruijin testing machine Co. Ltd., Jinan, China) after gelation. The relationship between uniaxial compressive strength and strain can be measured by setting different loading rates, and exporting them to a computer. Experimental materials were cylinder gel samples with size of φ25 × 50 mm (5% bentonite and 10.3% XNGJ-3, 10% polymer gel particles, and 0.3% of bridge material of the modification fiber). The uniaxial compression apparatus and the cylinder gel samples are shown in Figure 10.

#### 3.3.2. Experiment Results

**Figure 11:**
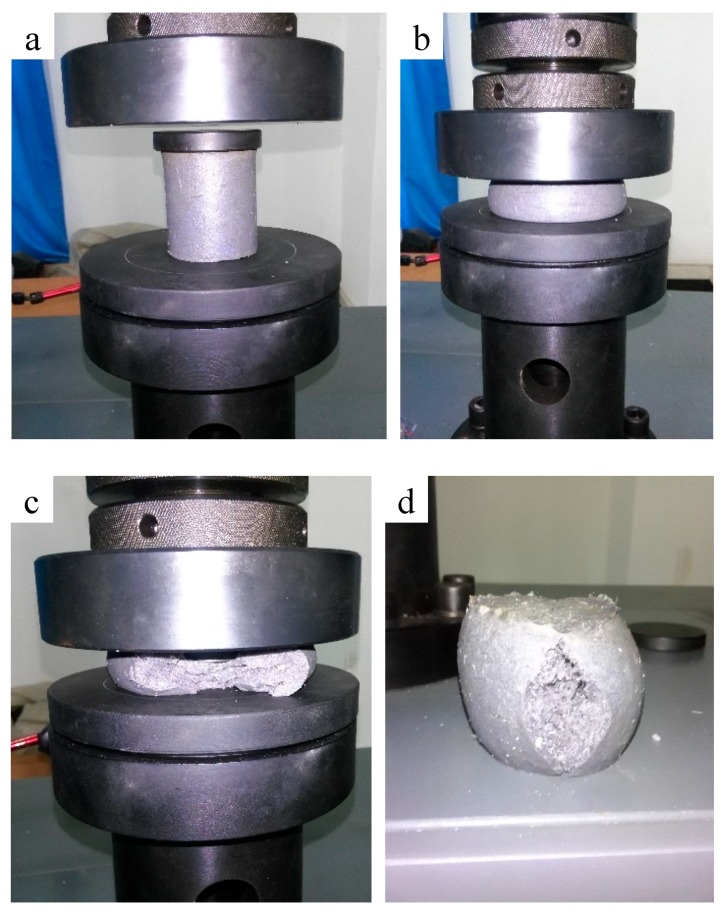
The deformation process of the new materials: (**a**) initial material; (**b**) undamaged material under high pressure; (**c**) failure material under high pressure; (**d**) recovery of material after pressure relief.

**Figure 12:**
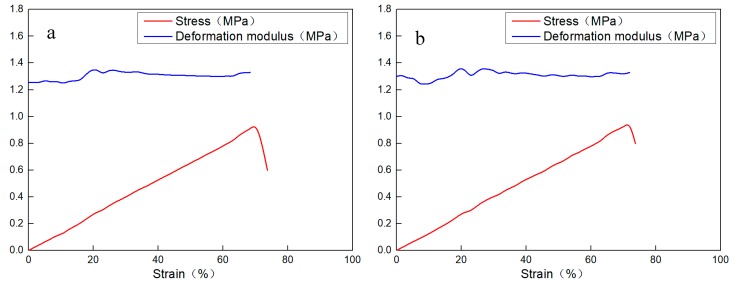
The stress–strain curves of the new material. (**a**) The curve of sample 1; (**b**) the curve of sample 2.

The material has great deformation at the given pressure without being damaged, as shown in Figure 11. Figure 12 is the stress–strain curves of the new material. The mechanical property parameters of the new material after gelation are in Table 3.

The following conclusions can be drawn from the experimental results. (1) The material has a high deformation and failure strain that can reach 70%. (2) During the elastic deformation stage, there is a clear linear positive correlation between the stress and strain. (3) After being pressed open, the material is still joined in the form of gel blocks and has a strong deformation recovery ability. These characteristics indicate that the gel has a good deformability which means it can avoid being damaged during the process of fracture closure.

### 3.4. Dynamic Plugging Simulating Experiment

In order to evaluate the plugging effect of XNGJ-3, a dynamic plugging simulation experiment was carried out based on actual working conditions, which is introduced as follows.

#### 3.4.1. Experiment Device

**Figure 13:**
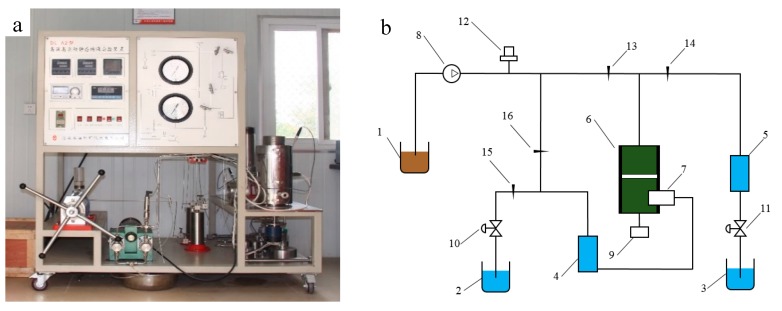
The high temperature and pressure dynamic simulator: (**a**) A picture of the real product. (**b**) The structure diagram, in which 1, 2, and 3 are vessels; 4 and 5 are piston vessels; 6 is a reaction vessel; 7 is a simulating fracture which is composed of two semi-columns with a length 5 cm and of adjustable width, as shown in Figure 14; 8 is an advection pump which works with pressures that can reach 40 MPa and a flow rate if 0.01 to 20 mL/min; 9 is an electric motor; 10 and 11 are safety valves; 12 us a pressure transducer; and 13, 14, 15, and 16 are valves.

The dynamic plugging simulating experiment aimed to simulate the plugging state of the plugging material under the formation environment and then judge the plugging quality, which can provide scientific data for practical applications. A high temperature and pressure dynamic plugging experimental device No. DL-A2, manufactured by Haian Petroleum Technologies Co. Ltd. (Haian, China) was used to evaluate the plugging effect, as shown in Figure 13.

#### 3.4.2. Experiment Method

There are three stages to simulate the dynamic plugging process, as shown below.

**Figure 14:**
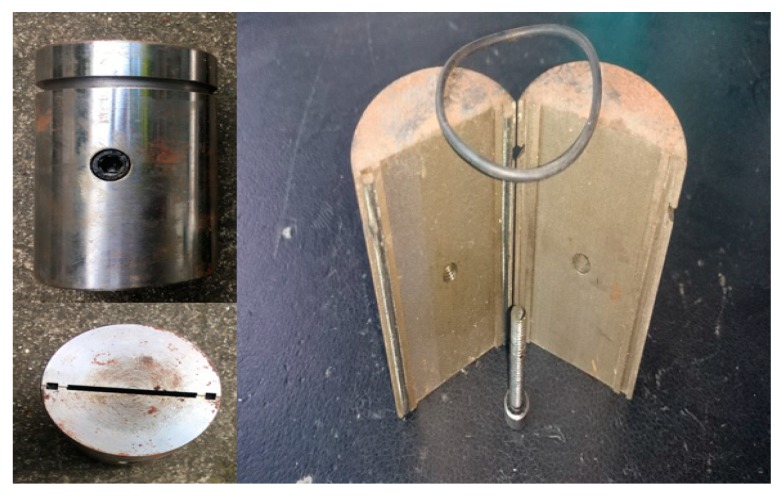
The fracture simulating component.

Stage 1: All valves should be closed, then the fracture simulating component (Figure 14) was closed, and the formation fluid (tap water in this experimental) was poured into piston vessels 4 and 5. Following this, the plugging material was put into the reaction vessel and was stirred by an electric motor until it was intensively mixed. This stage aims to simulate the transfer process of the plugging material in the wellbore before it reaches the fracture.

Stage 2: The second stage begins at the end of the agitation, then valves 13 and 15 were opened and the drilling fluid was poured from vessel 1 into 6 by an advection pump under 1.5 MPa. In this stage, the plugging material did not gel and was able to flow into the strata under the simulative pressure difference between the wellbore and the formation.

Stage 3: The third stage started when the fluid in vessel 2 was kept constant, which indicated that the fracture was blocked. Then, we took out the simulating fracture component and cleaned all the material outside the fracture (the plugging material outside the crack would be removed with the drilling fluid cycle in the actual situation). Following this, the simulation fracture component was fixed again and the drilling fluid was poured into reaction vessel 6. The bearing capacity of the plugging material was tested by the pressure offered by the advection pump connected by the drilling fluid. So, the plugging material would gel under high temperature and pressure. Meanwhile, the inverse pressure would be tested by opening vessels 14 and 16, and closing vessels 13 and 15. This stage tests the bearing capacity of the plugging material.

According to standard engineering practice, 5% bentonite + 10.3% XNGJ-3 (10% polymer gel particles and 0.3% bridge material of the modification fiber) were mixed with water to form the sample. The poly-sulfur high-density drilling fluid was used to test the plugging ability of the gel, which had a density of 2.0 g/cm^3^, a viscosity of 32 mPa·s, and a thickener coefficient of 0.85 Pa·s.

#### 3.4.3. Experiment Results of the Bearing Capacity

Table 4 presents the results of the dynamic plugging simulating experiment under temperatures of 80 °C, 90 °C, 105 °C, and 120 °C and fractures widths of 2 mm, 3 mm, 4 mm, and 5 mm, respectively. Table 2 shows that XNGJ-3 can plug the fractures with widths of 2 mm, 3 mm, 4 mm, and 5 mm, which implies a good plugging effect. Table 5 shows the comparison of the plugging effect between XNGJ-3 and the other plugging gels. The comparison results in Table 5, which shows that the bearing capacity of XNGJ-3, which is more than 20 MPa, is nearly twice as large as the other three kinds of plugging materials. The inverse bearing capacity of XNGJ-3 is 20 MPa under different conditions; the high inverse bearing capacity can avoid the plugging material flowing back into the wellbore. These results imply that XNGJ-3 has a good advantage to plug the fracture formation compared to several other plugging materials.

#### 3.4.4. Experimental Results of the Leakage

**Figure 15:**
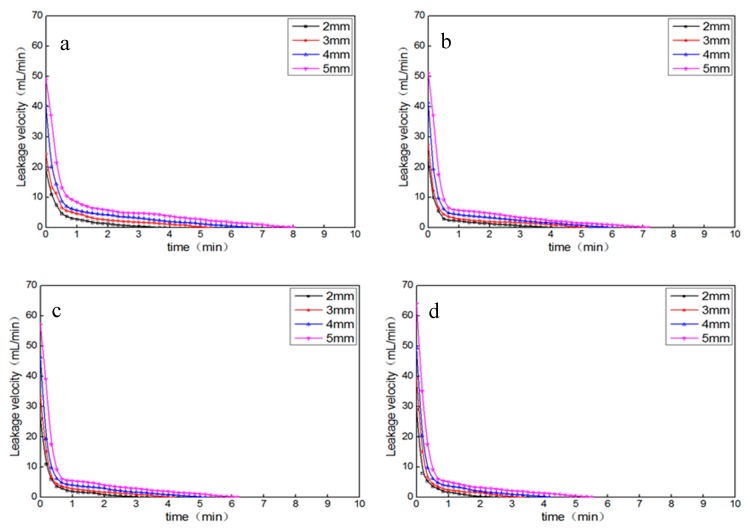
The relationship between the leakage velocity and time: (**a**) at 80 °C; (**b**) at 90 °C; (**c**) at 105 °C; (**d**) at 120 °C. The starting point of time is the point of time that the material began to leak.

In order to analyze the leakage factors in the plugging process, the leakage was recorded every five seconds in the second stage, and the leakage velocity curve was drawn as shown in Figure 15. The following results can be clearly seen from Figure 15. (1) The initial leakage velocity is high and then reduces rapidly, and the leakage velocity and time have a good exponential function relationship. (2) Under the same temperature, the initial leakage velocity increases and the leakage stopping time grows with increase in the fracture width. (3) For the same fracture width, the initial leakage velocity increases and the leakage stopping time reduces with the increase in temperature. The reason for these is that XNGJ-3 is easier to flow, and the whole coagulation time is shorter at a higher temperature, which coincides with the fourth result of the thickening performance test.

### 3.5. Summary of the Material’s Properties 

Through these experimental analyses, the main properties of the material are as follows. (1) The material is used as a gel solvent with a good fluidity and can enter fractures smoothly in the actual plugging process. (2) The gel solvent cannot form a gel solid under 80 °C, but when the temperature reached 80 °C, it gelled, and the gelling time was controlled within the required time of the practical application. (3) The new material has a high deformation which can avoid being damaged during the process of fracture closure. (4) From the dynamic plugging simulating experiment, the bearing capacity of XNGJ-3 can reach 21 MPa and its inverse bearing capacity can reach 20 MPa.

Most plugging gels are preformed gels and sent to the strata in a gel state. If a gel has a high strength, it is not able to enter small fractures. If a gel has a low strength, it is not able to form a stable plugging layer. XNGJ-3 is a soluble polymer gel. It can be sent to the strata in a gel liquid and polymerized with a high strength. In order to illustrate the advantages of XNGJ-3, the material is compared with bridge plugging material and common plugging gel in Table 6.

From Table 6, we found that performance of XNGJ-3 is better than the others during plugging the well leakage in middle-deep complex fractured strata. Although, since acrylamide monomers are supposed to have carcinogenicity or toxicity, there are strict regulations and comprehensive protective measures in the production, storage, and use of materials. The pollution of the material to the environment is negligible because the material is used thousands of meters below the surface. From the perspective of fast and effective plugging of XNGJ-3, the new material is applicable to the deep strata environment because a large number of drilling fluid leakage incidents will increase strata pollution. After a series of experimental evaluations and comparative analyses, XNGJ-3 has been certified to be effective for plugging fractured strata.

## 4. Engineering Applications of XNGJ-3

### 4.1. Leakage Characteristics of the Study Area

The Sichuan basin, where there are abundant oil and gas resources, is an important area for oil and gas exploitation in southwest China. However, most of the wellbores have well leakage problems. To deal with these problems, various kinds of plugging methods, including plugging while drilling, bridge plugging, well cementation plugging, and so on, have been used in this area, but all of them have had low success ratios for the first plugging. According to incomplete statistical data, in the Xujiahe formation, the average plugging times for every leakage well was 6.4 and the successful ratio of the first plugging was only 20%. In the Yuanba continental formation, the average plugging time of every leakage well was 11, and the success ratio of the first plugging was only 25%.

**Figure 16:**
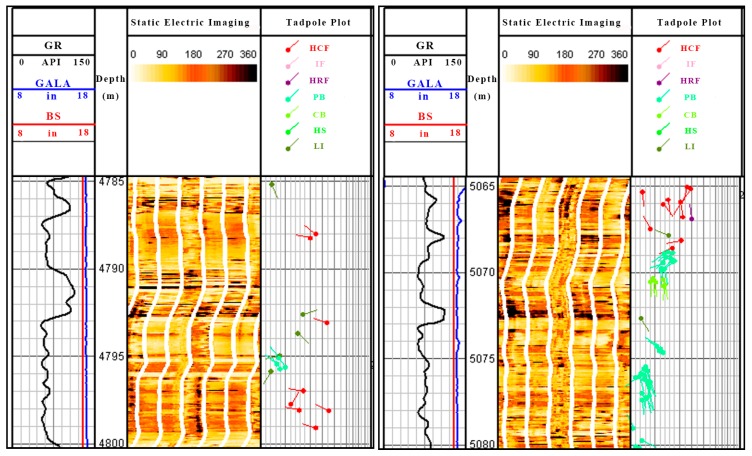
The logging image of Well X1 in the Sichuan west area: HCF indicates the high conductive fractures, IF indicates the induced fractures, HRF indicates the high resistant fractures, PB indicates the parallel bedding, CB indicates the cross bedding, HS indicates the horizontal stratification, LI indicates the layer interface, and SS indicates the surface of the scour.

**Figure 17:**
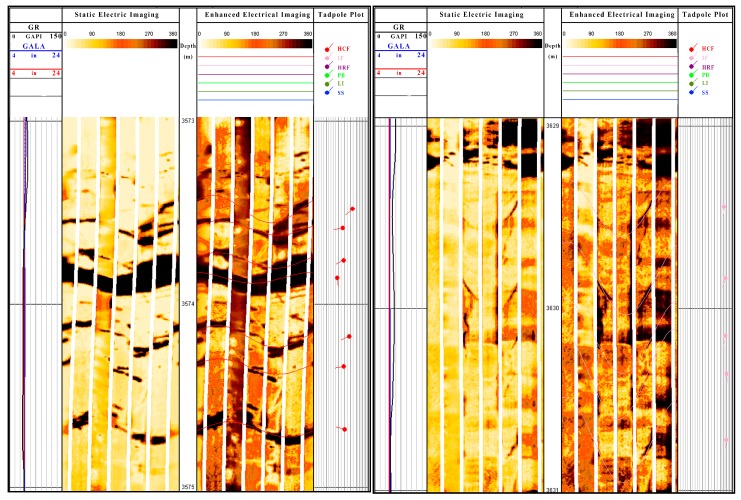
The logging image of Well D1 in the Sichuan east area.

Figure 16 and Figure 17 present the logging images of Well X1 in the Sichuan west area and Well D1 in the Sichuan east area. According to the log data, the strata bedding plane is complicated and has a large fracture density and the fracture extends crossly, which causes fragile rocks and frequent leakages which are hard to plug.

### 4.2. Engineering Application Description

Three leakage wells ZY3, X502, and ZJ107 were used to study the engineering applications of XNGJ-3. Table 7 describes the plugging effect of the three leakage wells. The composition proportion of the plugging material was determined according to the temperature and pressure of formation. 

The well leakages of Well ZY3 and Well X502 were both caused by strata natural fractures and were barely plugged by normal plugging methods. However, as shown in Table 7, the leakages of the three wells were successfully controlled using XNGJ-3,which revealed that this plugging material had effective plugging properties. The leakage of Well ZJ107, which was caused by improper operations, was also controlled by XNGJ-3, and this well did not leak in the subsequent operation. Both of kinds of well leakages have been plugged successfully, which indicated that XNGJ-3 can meet the requirements of standard engineering practice.

### 4.3. Plugging Evaluation

Table 8 presents the plugging evaluation of XNGJ-3 compared to the normal plugging materials. Firstly, for the three wells ZY3, X501, and ZI107, which were all drilled through the fractured strata, the plugging success rate was 100% at first plugging, which is much higher than normal plugging materials. Secondly, although the one-time cost of XNGJ-3 is higher than that of the normal plugging material, the total cost of the former may be much lower than the latter, considering the cost of the drilling fluid leakage and plugging time. Thirdly, more advantages of XNGJ-3 if we take safe operation and harm of leakage to the strata into consideration. 

From what have discussed above, it has been proven that XNGJ-3 can increase the strata bearing capacity in the fractured strata, which can greatly increase the success ratio. XNGJ-3 can also decrease the drilling cost and the drilling time and enhance the well-control capacity. All in all, lower cost and the more downhole safety will be obtained if XNGJ-3 is used to solve well leakages caused by fractured strata.

## 5. Conclusions

Well leakage accidents caused by fractured strata are a major downhole problem that is barely resolved during wellbore drilling. This unwieldy problem, caused by the poor formation of the cementing degree, the staggered-mesh of the fracture, and the low bearing capacity of the formation, can lead to the narrow and even unsafe window of drilling fluid density. In order to improve the plugging effect of the fractured strata, we developed a polymer plugging gel, named XNGJ-3, and the performance and application were also measured and evaluated. Some conclusions were drawn as follows:

1. Under the nitrogen environment, acrylamide and the monomers containing carboxyl and hydroxyl were reacted together to form the gel. This gel (XNGJ-3) was able to form a kind of gel particle after drying, grinding, and screening. Using these gel particles, a polymer plugging gel (XNGJ-3) was obtained by adding a bridge material of the hydrophilic fiber.
2. According to the results of the fluidity test, the plastic viscosity, dynamic shearing force and static shearing force of the gel solvent were very low within 3 h after preparation (this is enough time for the other preparations before plugging). According to the results of coagulation time test, when the temperature reached 80 °C, XNGJ-3 gelled, and the gelling time was controlled within the required time of the practical application; these properties are beneficial for making the plugging material enter the crossing fracture smoothly and occlude the fracture.
3. According to the uniaxial compression test, the new material has a high deformation and failure strain that can reach 70%. It also has clear linear positive correlation between stress and strain. After being pressed open, the material is still joined in the form of gel blocks and has strong deformation recovery ability. These characteristics indicate that the gel has a good deformability which can avoid being damaged during the process of fracture closure.
4. The bearing capacity of XNGJ-3 can reach 21 MPa and its inverse bearing capacity can reach 20 MPa, which is much higher than other plugging gels. This is good for the stability of the plugging layer in subsequent construction. The gel can plug quickly and effectively, which helps to reduce leakage and costs in the process of plugging. It also reduces the damage of drilling fluid leakage to strata.
5. According to the application evaluation of the three leakage wells with the different leakage characteristics, well leakage can be well controlled after using XNGJ-3 and will not happen again after plugging. Compared with common plugging materials, XNGJ-3 has a lot of advantages including better economic benefits, less plugging time cost, and higher plugging success ratio for the fractured strata, which implies that XNGJ-3 has a good application value for fractured strata.

## Figures and Tables

**Table 1:** The fluidity test results.

Time after Preparation (min)	Group Number	Φ600 /Φ300	Φ200 /Φ100	Φ6 /Φ3	Plastic Viscosity (mPa·s)	Dynamic Shearing Force (Pa)	Static Shearing Force (Pa)
0	1	25.5/15.5	12.5/9.5	6.5/6	10	2.8105	3.066
	2	25/15.5	12.5/9.5	6.5/5.5	9.5	3.066	2.8105
60	1	25.5/16	13/9.5	7.5/7	10.5	3.3215	3.577
	2	25/16	12.5/10	7.5/7	10	3.577	3.577
120	1	27/16.5	13.5/9.5	7/7	11.5	3.066	3.577
	2	27/16.5	13/11	7/6.5	11	3.066	3.3215
180	1	29/17.5	14/9.5	9/7.5	13	3.066	3.8325
	2	29.5/18	14/11.5	8.5/8	13.5	3.3215	4.088

**Table 2:** The test conditions and the results for evaluating the coagulation time of XNGJ-3.

Experiment Condition	Coagulant (%)	Retarder (%)	Primary Congeal Time (min)	Hardening Time (min)	Consistency Curves
70 °C, 20 MPa	2	0	Not congealed	-	Figure 8a
80 °C, 50 MPa	1	0	154	15	Figure 8b
90 °C, 50 MPa	0	0	144	14	Figure 8c
105 °C, 65 MPa	0	0.2	60	9	Figure 8d
	0	0.4	64	8	Figure 8e
	0	0.6	124	10	Figure 8f
120 °C, 80 MPa	0	0.6	58	4	Figure 8g
	0	0.8	97	5	Figure 8h

**Table 3:** The mechanical property parameters of the material after gelation.

Number	Diameter (mm)	Length (mm)	Compressive Strength (MPa)	Elastic Modulus (Mpa)	Strain at Failure Point (%)	Pressure-Deformation Relationship
1	45.55	57.55	0.94	1.35	69.5	Figure 12a
2	45.60	56.45	0.96	1.32	72.6	Figure 12b

**Table 4:** The results of the dynamic plugging simulating experiment.

Temperature (°C)	Retarder (%)	Coagulant (%)	Stirring Time (min)	Fracture Size (mm)	Simulation Pressure Difference (MPa)	Leaking Amount (mL)	Forward Bearing Pressure (MPa)	Inverse Bearing Pressure (MPa)
80	0	1	140	2	1.5	50	21	20
				3	1.5	61	21	20
				4	1.5	104	21	20
				5	1.5	123	21	20
90	0	0	130	2	1.5	50	21	20
				3	1.5	60	21	20
				4	1.5	98	21	20
				5	1.5	120	21	20
105	0.6	0	110	2	1.5	49	21	20
				3	1.5	59	21	20
				4	1.5	95	21	20
				5	1.5	112	21	20
120	0.8	0	80	2	1.5	48	23	20
				3	1.5	59	23	20
				4	1.5	81	21	20
				5	1.5	94	21	20

**Table 5:** The comparison of the plugging effect between XNGJ-3 and other plugging materials.

Material Name	Test Descriptions	Test Results
Polyacrylamide gel (GPAM) [31]	Formulation of plugging material: 300 mL basic slurry + 100 mL GPAM + ocl-BST-1. Test conditions: sand bed dehydration at 25 °C.	The bearing capacity is 2.25 MPa with a granularity between 0.18 and 0.28 mm of sand samples.
Composite chemical gel (OCL-GYDL) [32]	Formulation of plugging material: 6% solid flour basic slurry + 3% OCL-GYDL and 6% solid flour basic slurry + 5% OCL-GYDL. Test conditions: high temperature and pressure filter press.	The bearing capacity is 10 MPa with a granularity between 0.18 and 0.28 mm of sand samples.
Thermoresponsive Temporary Plugging Agent (SDA-8) [19,20]	Formulation of plugging material: SDA-8. Test conditions: artificial rock (5 cm × 2.54 cm), fracture width is 0.5 mm, and temperature of 105 °C.	The bearing capacity is 6.8 MPa.
XNGJ-3	Formulation of plugging material: 10.3% XNGJ-3 (10% polymer gel particles and 0.3% bridge material of the modification fiber) + reagent. Test conditions: high temperature and pressure test instruments, the test temperature was between 80 °C and 120 °C, and the fracture width was between 2 mm and 5 mm.	The bearing capacity is 21 MPa and the inverse bearing capacity can reach 20 MPa.

**Table 6:** Comparison between bridge plugging materials, common plugging gel, and XNGJ-3.

Contrastive Term	Bridge Plugging Material	Common Plugging Gel	XNGJ-3
State on the ground	Particles, lines, and flake solids with different sizes	Gel block	Gel solution
State in the fracture	A dense accumulation body with a small pore	Gel block	Gel block
Plugging mechanism	Large particles cannot pass the plugging layer and the permeability of the plugging layer is low.	Forming a slug that isolates drilling fluid and a leakage layer.	Forming a slug that isolates drilling fluid and a leakage layer.
Suitable for which fracture width	The material is suitable for fracture width of less than 5 mm.	The material is suitable for larger fractures, it is difficult for it to enter cracks with a width less than 2 mm.	The applicable fracture width of the material is wide.
Bearing capacity	High pressure capacity	General pressure capacity	High pressure capacity
Advantages	The material has a high bearing capacity and can plug general leakage.	The material has a good compatibility and can be used together with other materials to plug large fractures.	The material can be used in complex fractured strata and has a high bearing capacity.
Disadvantages	The application results in large fractures and complex pressure systems are poor.	The material is not suitable for small fractures and cannot be used alone to improve formation pressure.	The material cannot be used for gelation at temperature below 80 °C so it is not suitable for shallow strata.

**Table 7:** The plugging description of the three leakage wells.

Well Name	ZY3	X502	ZJ107
Location	He Ba syncline structure on Qian Jiang sag of southeast Chong Qing.	Northwest wing of Sichuan basin in the west Sichuan depression structure.	West wing of Sichuan basin in the west Sichuan depression Zhong Jiang structure.
Leakage features	9 leakages happened from 1441 m to 2054 m, leakage speed was from 1.5 m^3^/h to 12.7 m^3^/h. Both plugging while drilling and bridge plugging were invalidated, and the leakage amount was 325.71 m^3^.	The leakage happened when drilling down to 3113 m. Both plugging while drilling and bridge plugging did not work well. The plugging material returned during plugging, and the leakage amount was 255.73 m^3^.	After running the casing of the second open, the well leakage happened when the emission of the pump circulation increased to 28 L/s and the leakage velocity was 12.5 m^3^/h.
Reason Analysis	There are many strata fractures in the Xiaoheba and Longmaxi group and the bearing capacity of the formation is low.	According to log data and the results of the rock test, it was defined as the fractured leakage of the sandy gas layer bedding.	Because too many centralizers were installed in the well, the annulus was blocked by the rock debris, and the pressure of the annular space was higher than the bearing capacity of the formation.
Plugging Evaluation	There was no leakage after double plugging, one was at 2053.4 m and the other was before cannula sealing. The plugging material was made by 5% bentonite + 10.3% XNGJ-3 (10% polymer gel particles and 0.3% bridge material of the modification fiber).	The well has never leaked and the material has never returned again after plugging. The plugging material was made by 5% bentonite + 10.3% XNGJ-3 (10% polymer gel particles and 0.3% bridge material of the modification fiber).	Leakage was controlled and there was no leakage again when the emission increased in the normal range. The plugging material was made by 5% bentonite + 10.3% XNGJ-3 (10% polymer gel particles and 0.3% bridge material of the modification fiber).

**Table 8:** The plugging evaluation of XNGJ-3.

Well Number		ZY3	X502	ZJ107
Well depth (m)		756–2145	3113–3197	2842
Normal plugging material	Plugging times	9	5	0
	Material cost of single plugging (ten thousand CNY)	0.4	0.5	0
	Leakage of drilling fluid (m^3^)	325.71	359.12	0
	Leaking drilling fluid expense (ten thousand CNY)	57.9	71.2	0
	Plugging time (h)	65	89	0
	Cost of time expense (ten thousand CNY)	27.3	37.38	0
	Success ratio	33%	0	-
New plugging material mixed with XNGJ-3	Plugging times	2	1	1
	Material cost of single plugging (ten thousand CNY)	1.7	1.7	1.9
	Leakage of drilling fluid (m^3^)	20	15	11
	Leaking drilling fluid expense (ten thousand CNY)	3.57	2.97	1.76
	Plugging time (h)	16	5.5	4.5
	Cost of time expense (ten thousand CNY)	6.72	2.31	1.89
	Success ratio	100%	100%	100%
